# Sneezing in response to naturalistic bright light exposure

**DOI:** 10.12688/f1000research.167964.1

**Published:** 2025-10-17

**Authors:** Lucien Bickerstaff, Josef Trinkl, Stephan Munkwitz, Manuel Spitschan

**Affiliations:** 1 Technical University of Munich, Munich, Germany; 2 Max Planck Institute for Biological Cybernetics, Tübingen, Germany

**Keywords:** lhotic sneeze reflex, light-induced sneezing, bright light exposure, environmental lighting, sneezing reflex, reflex sensitivity, illuminance, pupillometry, nonvisual light effects, light-triggered sensations, real-world light logging, visual stimuli, multi-primary LED, integrating sphere, reflex threshold

## Abstract

**Background:**

The photic sneeze reflex (PSR) is a common but underexplored phenomenon where bright light triggers sneezing, affecting ~30% of the population. This study aimed to characterise the light conditions inducing PSR.

**Methods:**

One male PSR-affected participant logged sneezing events during a 30-day real-world light exposure study. An indoor setup using a multi-primary LED source and an integrating sphere delivered 30-second light stimuli while pupillometric data were collected.

**Results:**

A total of 82 sneezing events were recorded, averaging 2.73 sneezes/day (range: 1–6 per event). Illuminance increased tenfold before sneezing, peaking 2 minutes prior, and returned to baseline within 10 minutes. Despite exposure to 150+ stimuli, artificial sneezing was not induced, though high illuminance consistently triggered tickling sensations.

**Conclusions:**

Sudden increases in environmental lighting can provoke PSR. While artificial stimuli elicited only tickling, further refinement of the protocol could enable reliable PSR induction, thereby facilitating mechanistic research.

## Key points

• The manuscript presents a **case study** investigating the photic sneeze reflex (PSR), a common but underexplored phenomenon triggered by bright light exposure.
• It combines **real-world light exposure measurements** with **controlled laboratory experiments**, offering a detailed characterization of the conditions that elicit PSR.
• The findings highlight the role of **sudden changes in illuminance** as a trigger for PSR and propose improvements for experimental protocols to enable further mechanistic research.
• This case study provides a foundation for future investigations into the PSR and their clinical and physiological significance.

## Background

Photic sneezing is a widespread phenomenon, characterised by sneezing in response to bright light exposure (typically direct sunlight), reportedly affecting up to around 20-30% of the population ( Askenasy, 1990; Dean, 2012; Everett, 1964; Kulas et al., 2017; McKusick, 2003; Morris, 1987). The photic sneeze reflex (PSR) has been documented for decades ( Askenasy, 1990; Féré, 1890; Sédan, 1954), if not centuries, but despite its relatively high prevalence, is poorly understood. Some studies have attempted to further clarify the genetic and neural mechanisms involved in the reflex ( Breitenbach et al., 1993; Chowdhury et al., 2019; Collie et al., 1978; Hydén & Arlinger, 2009; Langer et al., 2010; Peroutka & Peroutka, 1984; Sasayama et al., 2018), leading to no conclusive results. There is at present no reliable in-laboratory stimulus to induce photic sneezing ( Trinkl et al., 2025).

To understand the naturalistic antecedents of the PSR, we examined sneezing in response to bright light exposure under naturalistic conditions while a photic sneezer logged their sneezes. In addition, we characterised tickle sensations and pupil responses to bright light exposure using white-light stimuli varying parametrically in illuminance.

## Methods

### Real-world light exposure measurements and photic sneeze logging

Real-life, daytime light measurements were carried out in summer between 12 July 2022 and 10 August 2022 (30 days) in and around Tübingen, Germany, as the participant went about his daily life. The participant, a healthy 21-year-old male (author LB of this manuscript), followed a Monday to Friday, 9-to-5 work schedule, working mostly indoors at a desk, at times with no natural light at all (only artificial light). Measurements were continuous from approximately 08:00 to 21:00, with a sampling frequency of 30 s. An ActTrust2 wearable actigraph with light logger (Condor Instruments, São Paolo, Brazil) was worn as a necklace so it would rest on the torso facing forwards. This provided a good compromise between accurate measurements (i.e., in the same direction as the corneal plane) and practical comfort. The device was fixed to the torso using a magnetic plate resting between the participant’s torso and their clothing, to prevent unwanted tilt or flipping.

Every time the PSR manifested, the participant would self-report the sneeze event in a sneeze log datasheet implemented on Notion. Each entry contain the precise date and time of the sneeze (to the minute) and the number of sneezes for that event, since one sneeze event can contain multiple sneezes.

### Analysis of light exposure and PSR event data

The precise logging of each sneeze event allowed to obtain the average light exposure levels 20 min before and after the event. This was then compared to a reference – measured to be the average illuminance over 40-min, randomly-chosen time windows (n = 100), when no sneeze event was reported. Individual contrasts between light exposure before and at each sneeze event were also analysed. Pre-sneeze light levels were averaged in time windows of 5 to 2 min before the sneeze event, and sneeze light levels in time windows ranging from 1 min before to 1 min after the sneeze event.

### PSR induction in controlled conditions

A custom setup was used to elicit the PSR under controlled laboratory conditions. While wearing a head-mounted eye tracker for pupillometry (Pupil Core, Pupil Labs, Berlin, Germany), the participant was exposed to bright light emitted by a 10-primary light source (Spectra Tune Lab, Ledmotive, Barcelona, Spain) light into an integrating sphere.

Two paradigms were used to elicit the PSR:

1. A “one-shot” paradigm, where the participant would follow a 10-minute dark adaptation period, and then be exposed to a single light stimulus lasting 30 seconds;
2. And a 30-minute paradigm, where the protocol was identical but instead of one single stimulus, 24 stimuli were presented to the participant in succession, with 60 seconds of refractory darkness between the stimuli.

The photopic illuminance of the light stimulation varied between four different pre-set illuminance settings, measured at approximately 440, 1100, 4400 and 17600 lx (photopic) at eye-level using a calibrated spectroradiometer (STS-VIS, Ocean Optics, Ostfildern, Germany). An additional dark setting (0 lx) was included in the 30-min experiment.

In addition to pupillometry, self-reports of sneeze onset (yes/no) and tickling sensation ratings from 0 (no tickle at all) to 10 (strong enough tickle to induce a sneeze) were obtained.

## Results

### Real-life light measurement and photic sneeze logging

**Figure 1.**
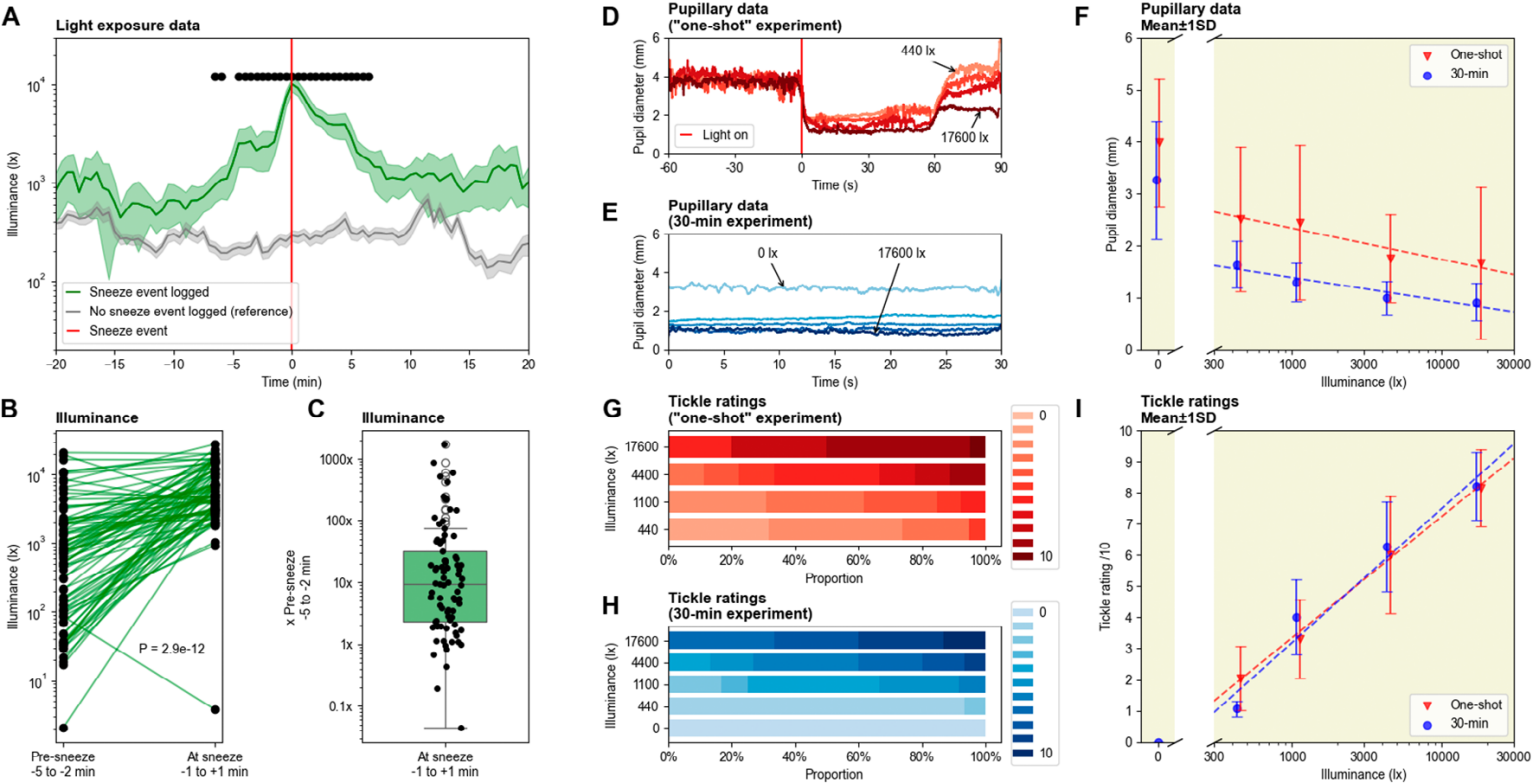
(A) Average light exposure 20 minutes before and after the sneeze event (n = 82). Reference is the average light exposure over randomly-chosen 40-minute windows, in which no sneeze event was recorded (n = 100). The light sensor was worn for 30 days during daytime (around 08:00 to 21:00), while all sneeze events were logged in parallel. Shaded error bars show standard error of the mean (SEM) and black dots show significance (independent t-test, p < .000610 after Bonferroni correction). (B, C) Difference between light exposure before (-5 to -2 min) and during (±1 min) sneeze events (linked t-test, t(81) = -8.21, p = 2.9e-12). (D) Average pupil diameter before and after light onset, for the one-shot paradigm. (E) Average pupil diameter during the 30-second light stimulations, for the 30-minute paradigm. (F) Comparison of the average pupil size as a function of illuminance for the two paradigms, mean ± STD. (G) Tickle ratings for the one-shot paradigm. (H) Tickle ratings for the 30-minute paradigm. (I) Comparison of the average tickle ratings as a function of illuminance for the two paradigms, mean ± STD.

Over the 30 day period, a total of 82 sneeze events were recorded, for an average of 2.73 sneezes per day. The number of sneezes range from 1 to 6 sneezes per event. Generally, a strong increase in light exposure can be observed in the few minutes leading to the sneeze event ( Figure 1). Only one instance of photic sneezing was reported when light levels decreased before the sneezing event occurred. More specifically, a 10-fold increase in light intensity can be noticed just before photic sneezing on average, with quartiles within 2× to 30×. The difference in light intensity is significant in the 20 minutes surrounding the sneeze event. Pre-sneeze illuminance ranges from around 20 lx to 20 000 lx, while light levels at sneeze events range from around 1 000 lx to almost 30 000 lx (excluding the outlying instance mentioned just above, where illuminance fell to around 40 lx).

After the sneeze event, the average illuminance typically falls back down to baseline levels within 10 minutes. The average light levels around photic sneezing events always remain above the reference, corresponding to the average illuminance for 100 time windows in which no photic sneeze was reported. Indeed, when sneezing was reported, the light levels almost always stayed above 500 lux, whereas when no sneezing was reported, they mostly remained below 500 lux. Sneezing often took place during transitions between environments, e.g., walking from home to the bus station, or from the bus station to the workplace.

### PSR induction in controlled conditions

In the experiments, we were unable to elicit a PSR using our laboratory stimuli. Despite exposure to more than 150 stimuli, the experimental setup could not induce sneezing in the participant. However, tickling sensations were consistently reported, and very high – although rare – values of 10/10 show that sneezing was very close under high light intensity. Both pupil and tickle ratings followed a monotonic relationship with the photopic illuminance of the stimuli.

## Conclusion

This study provides a detailed characterization of photic sneezing in a known photic sneezer. Real-world data showed that sudden increases in illuminance often precede photic sneezing. These findings reinforce the connection between rapid light intensity changes and the PSR, while also suggesting thresholds of illuminance that may be critical for its manifestation. Despite being unable to artificially elicit sneezes in a controlled setting, the strong tickling sensations observed suggest that the experimental paradigm is promising with further refinement. Future studies should investigate the variability in individual responses to artificial stimuli, and the underlying genetic, neurological and sensory mechanisms of the PSR. Additionally, expanding the study to a larger cohort will be essential to understand phenotypic variation and interindividual differences in the PSR.

## Ethics approval and consent to participate

This study was reviewed by the Ethics Committee of the Technical University of Munich (2024-74-W-SB). The participant gave informed consent in written form.

## Preprint

This manuscript was preprinted on bioRxiv ( Bickerstaff et al., 2024).

## Data Availability

All code and data are available:
• Data: https://doi.org/10.17617/3.LO8EXZ
○ Dataset for Bickerstaff et al., 2024 (DOI: 10.1101/2024.12.11.627890), containing pupillary data, subjective reporting data, light exposure and sneeze logging data and associated metadata. Data is provided in two forms: a full dataset containing all raw and exported data, and an exports-only dataset. • Code: https://github.com/tscnlab/BickerstaffEtAl_F1000Research_2025

○ Code repository for Bickerstaff et al., 2024 (DOI: 10.1101/2024.12.11.627890), containing a reproducible Python pipeline for generating the outcomes (analysis results and figures) presented in the manuscript.
