# Sneezing in response to naturalistic bright light exposure

**DOI:** 10.12688/f1000research.167964.2

**Published:** 2026-03-20

**Authors:** Lucien Bickerstaff, Josef Trinkl, Stephan Munkwitz, Manuel Spitschan

**Affiliations:** 1 Technical University of Munich, Munich, Germany; 2 Max Planck Institute for Biological Cybernetics, Tübingen, Germany

**Keywords:** lhotic sneeze reflex, light-induced sneezing, bright light exposure, environmental lighting, sneezing reflex, reflex sensitivity, illuminance, pupillometry, nonvisual light effects, light-triggered sensations, real-world light logging, visual stimuli, multi-primary LED, integrating sphere, reflex threshold

## Abstract

**Background:**

The photic sneeze reflex (PSR) is a common but underexplored phenomenon where bright light triggers sneezing, affecting ~30% of the population. This study aimed to characterise the light conditions inducing PSR.

**Methods:**

One male PSR-affected participant logged sneezing events during a 30-day real-world light exposure study. An indoor setup using a multi-primary LED source and an integrating sphere delivered 30-second light stimuli while pupillometric data were collected.

**Results:**

A total of 82 sneezing events were recorded, averaging 2.73 sneezes/day (range: 1–6 per event). Illuminance increased tenfold before sneezing, peaking 2 minutes prior, and returned to baseline within 10 minutes. Despite exposure to 150+ stimuli, artificial sneezing was not induced, though high illuminance consistently triggered tickling sensations.

**Conclusions:**

Sudden increases in environmental lighting can provoke PSR. While artificial stimuli elicited only tickling, further refinement of the protocol could enable reliable PSR induction, thereby facilitating mechanistic research.

## Key Points

• The manuscript presents a **case study** investigating the photic sneeze reflex (PSR), a common but underexplored phenomenon triggered by bright light exposure.
• It combines **real-world light exposure measurements** with **controlled laboratory experiments**, offering a detailed characterization of the conditions that elicit PSR.
• The findings highlight the role of **sudden changes in illuminance** as a trigger for PSR and propose improvements for experimental protocols to enable further mechanistic research.
• This case study provides a foundation for future investigations into the PSR and their clinical and physiological significance.

## Background

Photic sneezing is a widespread phenomenon, characterised by sneezing in response to bright light exposure (typically direct sunlight), reportedly affecting up to around 20-30% of the population ( Askenasy, 1990; Dean, 2012; Everett, 1964; Kulas et al., 2017; McKusick, 2003; Morris, 1987). The photic sneeze reflex (PSR) has been documented for decades ( Askenasy, 1990; Féré, 1890; Sédan, 1954), if not centuries, but despite its relatively high prevalence, is poorly understood. Some studies have attempted to further clarify the genetic and neural mechanisms involved in the reflex through experimental means, including EEG ( Breitenbach et al., 1993; Chowdhury et al., 2019; Collie et al., 1978; Hydén & Arlinger, 2009; Langer et al., 2010; Peroutka & Peroutka, 1984; Sasayama et al., 2018), though leading to no conclusive results. The pathophysiology of the reflex has long been subject to three theories ( Everett, 1964; Trinkl et al., 2025). The first is the optic-trigeminal summation theory, supposing a crosstalk between the second (CN II) and the fifth (CN V) cranial nerve at the level of the mid-brain. Second is the parasympathetic hypersensitivity theory, assuming a general hypersensitivity of the photic sneezers’ parasympathetic system. The third and final theory is mentioned as the parasympathetic generalisation theory, positing a co-activation of closely situated neural branches in the parasympathetic nervous system. All these theories may have neuroanatomical validity, however, so far, none have received validation through sufficient scientific evidence.

There is at present no reliable in-laboratory stimulus to induce photic sneezing ( Trinkl et al., 2025), which limits the potential to understand its mechanistic underpinnings.

To understand the naturalistic antecedents of the PSR, we examined sneezing in response to bright light exposure under naturalistic conditions while a photic sneezer logged their sneezes. In addition, we characterised tickle sensations and pupil responses to bright light exposure using white-light stimuli varying parametrically in illuminance.

## Methods

### Real-world light exposure measurements and photic sneeze logging

Real-life, daytime light measurements were carried out in summer between 12 July 2022 and 10 August 2022 (30 days) in and around Tübingen, Germany, as the participant went about his daily life. The delay between data collection and publication of the results is explained by intervening research internships, before returning to the project. The participant, a healthy 21-year male (author LB of this manuscript), followed a Mon-Fri, 9-to-5 work schedule, working mostly indoors at a desk, at times with no natural light at all (only artificial light). The participant did not wear any eye correction device at the time data was collected, and although no clinical evaluation by an ENT (ear, nose and throat) specialist, ophthalmologist, and/or neurologist was performed prior to inclusion, the participant does not report any relevant medical history that could influence PSR. Measurements were continuous from approximately 08:00 to 21:00, with a sampling frequency of 30 s. An ActTrust wearable actigraph with light logger (Condor Instruments, São Paolo, Brazil) was worn as a necklace so it would rest on the torso facing forwards. This would allow a good compromise between accurate measurements (i.e., in the same direction as the corneal plane) and practical comfort. The device was fixed to the torso using a magnetic plate resting between the participant’s torso and their clothing, to prevent unwanted tilt or flipping.

Every time the PSR manifested, the participant would self-report the sneeze event in a sneeze log datasheet implemented on Notion. Each entry contains the precise date and time of the sneeze (to the minute) and the number of sneezes for that event, since one sneeze event can contain multiple sneezes.

### Analysis of light exposure and PSR event data

The precise logging of each sneeze event allowed us to obtain the average light exposure levels 20 min before and after the event. This was then compared to a reference, defined as the average illuminance over 40-min, randomly-chosen time windows (n=100), when no sneeze event was reported. Individual contrasts between light exposure before and at each sneeze event were also analysed. Pre-sneeze light levels were averaged in time windows of 5 to 2 min before the sneeze event, and sneeze light levels in time windows ranging from 1 min before to 1 min after the sneeze event.

### PSR induction in controlled conditions

A custom setup was used to elicit the PSR under controlled laboratory conditions. While wearing a head-mounted eye tracker for pupillometry (Pupil Core, Pupil Labs, Berlin, Germany), the participant was exposed to bright light emitted by a 10-primary light source (Spectra Tune Lab, Ledmotive, Barcelona, Spain) light into an integrating sphere.

Two paradigms were used to elicit the PSR:

1. A “one-shot” paradigm, where the participant would follow a 10-minute dark adaptation period, and then be exposed to a single light stimulus lasting 30 seconds;
2. And a 30-minute paradigm, where the protocol was identical but instead of one single stimulus, 24 stimuli were presented to the participant in succession, with 60 seconds of refractory darkness between the stimuli.

The photopic illuminance of the light stimulation varied between four different pre-set illuminance settings, measured at approximately 440, 1100, 4400 and 17600 lx (photopic) at eye-level using a calibrated spectroradiometer (STS-VIS, Ocean Optics, Ostfildern, Germany). An additional dark setting (0 lx) was included in the 30-min experiment.

In addition to pupillometry, self-reports of sneeze onset (yes/no) and tickling sensation ratings from 0 (no tickle at all) to 10 (strong enough tickle to induce a sneeze) were obtained.

## Results

### Real-life light measurement and photic sneeze logging

**Figure 1.**
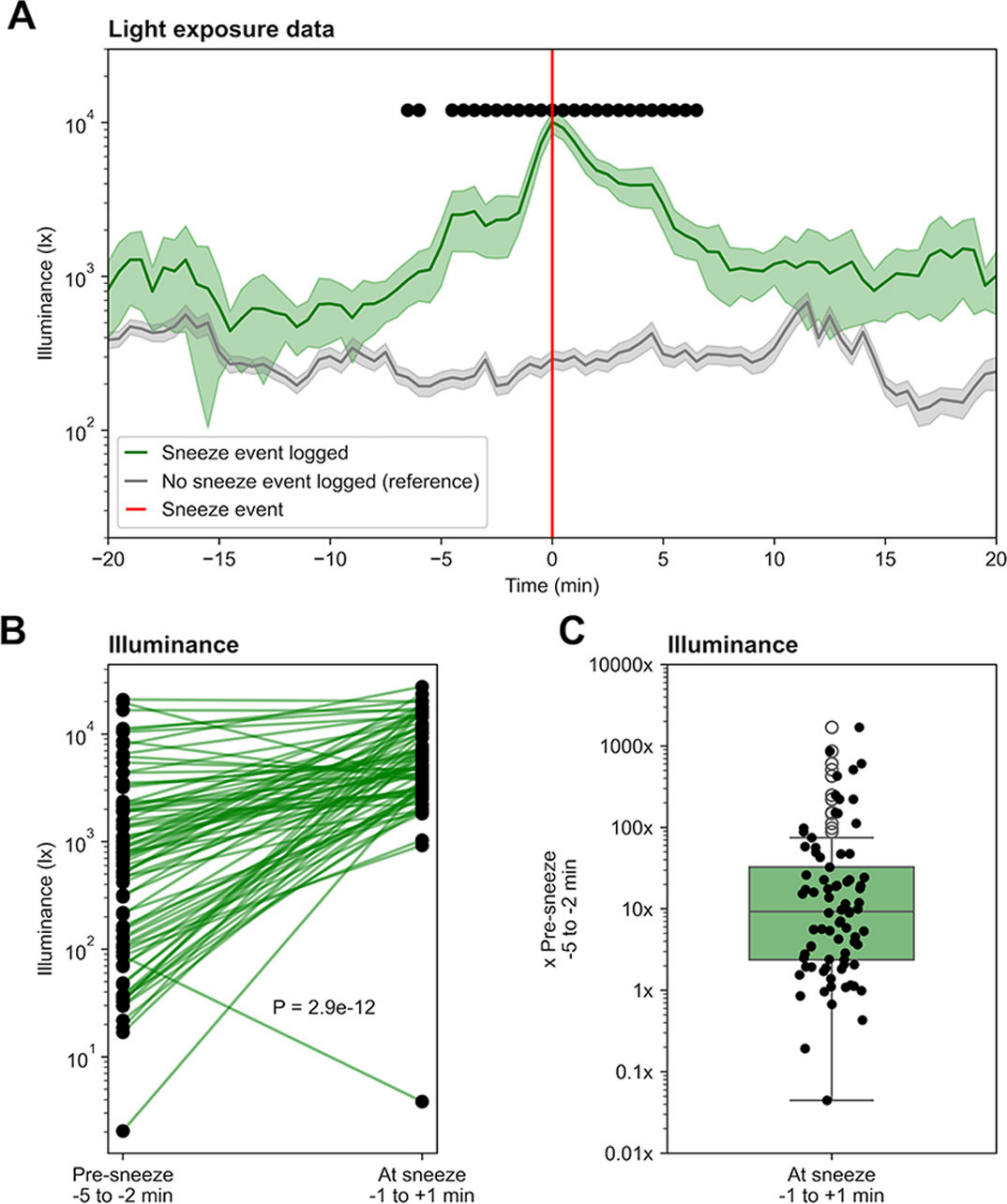
(A) Average light exposure 20 minutes before and after the sneeze event (n=82). Reference is the average light exposure over randomly chosen 40-minute windows, in which no sneeze event was recorded (n=100). The light sensor was worn for 30 days during daytime (around 08:00 to 21:00), while all sneeze events were logged in parallel. Shaded error bars show standard error of the mean (SEM) and black dots show significance (independent t-test, p < .000610 after Bonferroni correction). (B, C) Difference between light exposure before (-5 to -2 min) and during (±1 min) sneeze events (linked t-test, t(81) = -8.21, p = 2.9e-12).

Over the 30-day period, a total of 82 sneeze events were recorded, for an average of 2.73 sneezes per day. The number of sneezes ranges from 1 to 6 sneezes per event. Generally, a strong increase in light exposure can be observed in the few minutes leading to the sneeze event ( Figure 1A). A baseline is defined as the average illuminance for 100 randomly selected time windows in which no photic sneeze was reported. The change in light intensity is significantly different from this baseline in the 20 minutes surrounding the sneeze event (independent t-tests, p < .000610 after Bonferroni correction). The average light levels around photic sneezing events always remain above the baseline. Indeed, when sneezing was reported, the light levels almost always stayed above 500 lx, whereas when no sneezing was reported, they mostly remained below 500 lx. After the sneeze event, the average illuminance typically falls back down to baseline levels within 10 minutes.

Across all events, in the pre-sneeze window (-5 to -2 minutes before sneezing), the geometric mean illuminance was 527.3 lx (GSD = 7.2), whereas in the at-sneeze window (-1 to +1 minute around sneezing), the geometric mean illuminance was 5349.0 lx (GSD = 3.1) ( Figure 1B), which was determined to be a statistically significant difference after excluding one outlier (paired t-test, t(81) = -8.21, p = 2.9e-12). A 10-fold median increase in illuminance can be noticed between pre-sneeze and at-sneeze windows, and the mean log _10_-transformed ratio was 1.01 (SD = 0.86) ( Figure 1C). Pre-sneeze illuminance ranges from around 20 lx to 20,000 lx, while light levels at sneeze events range from around 1 000 lx to almost 30000 lx ( Figure 1B). 7/82 (8.5%) logs of photic sneezing were reported when illuminance decreased before the sneezing event occurred ( Figure 1B), with ratios of 0.04, 0.19, 0.43, 0.67, 0.85, 0.96 and 0.99 compared to pre-sneeze levels.

The participant noted that sneezing often took place during transitions in environment, i.e., walking from home to the bus station, or from the bus station to the workplace.

### PSR induction in controlled conditions

**Figure 2.**
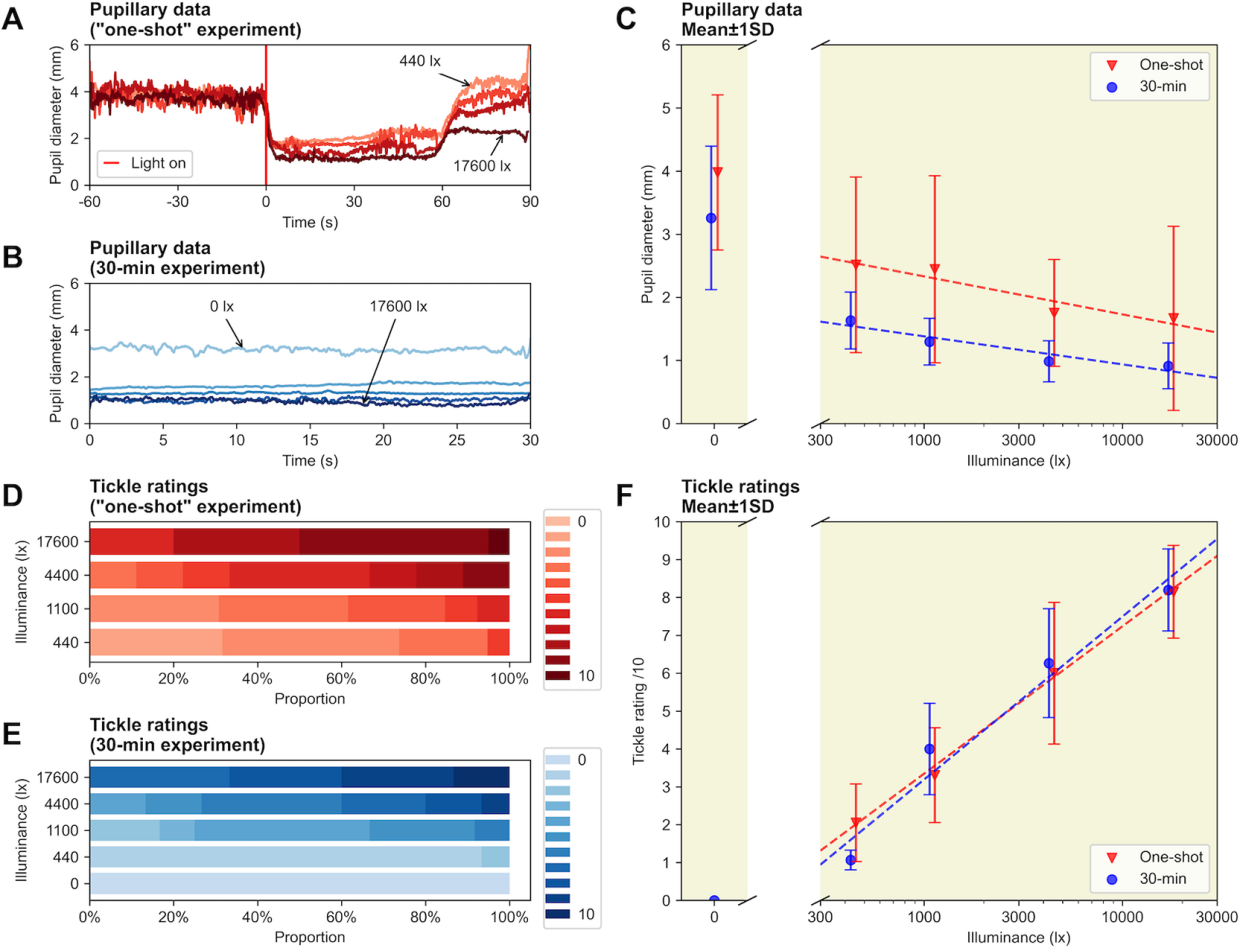
(A) Average pupil diameter before and after light onset, for the one-shot paradigm. (B) Average pupil diameter during the 30-second light stimulations, for the 30-minute paradigm. (C) Average pupil size as a function of illuminance for the two paradigms, mean ± STD. (D) Tickle ratings for the one-shot paradigm. (E) Tickle ratings for the 30-minute paradigm. (F) Average tickle ratings as a function of illuminance for the two paradigms, mean ± STD.

In the experiments, we were unable to elicit a PSR using our laboratory stimuli. Despite exposure to more than 150 stimuli, the experimental setup could induce not sneeze in the participant. However, tickling sensations were consistently reported, and very high – although rare – values of 10/10 show that sneezing was very close under high light intensity ( Figure 2D, E, F). Both pupil and tickle ratings followed a monotonic relationship with the photopic illuminance of the stimuli, with pupil constriction increasing with illuminance ( Figure 2A, B, C).

## Discussion and limitations

This study provides a detailed characterisation of photic sneezing in a known photic sneezer. As a case report, this study collected and analysed data from one participant only, which may mask responses observed in the general photic sneezing population.

Real-world data showed that sudden increases in illuminance often precede photic sneezing. These findings reinforce the connection between rapid light intensity changes and the PSR, while also suggesting typical thresholds of changes in illuminance that may be critical for its manifestation (10x). This is in line with the existing reports in the literature ( Trinkl et al., 2025), which however do not provide any quantitative information for comparison, e.g. PSR-inducing illuminance levels or thresholds – which this study does. 7/82 (8.5%) logs showed the opposite trend, showing a decrease in light intensity leading to sneezing. This could be due to the analysis approach, as binning the data into windows of the order of minutes can encapsulate periods of misrepresentative data, influencing the means. Indeed, it is very easy for the participant to block the light logger with a jacket, arm, or bag, decreasing light entering the light sensor. There is also a discrepancy between the position and directionality of the light sensor compared to the participant’s eyes – this might have led to incorrect measurements. It is reasonable to challenge the fact a decrease in measured illuminance would have triggered PSR events, though with generally strong enough light and help from (uncontrolled) environmental factors like dust or allergens in the air, it might have been possible such events were captured in the data.

Sneezing often took place during transitions in environment, i.e., walking from home to the bus station, or from the bus station to the workplace. This involves changes in lighting conditions, but not only – equally, temperature may change, and even though it was not measured here, it may also play a critical physiological role. The indoor temperature was mostly stable and comfortable, differing from the outdoor conditions when sneezing mostly occurred. Thermal instability is therefore a consideration, as sneezing and other nasal reflex responses may be triggered by exposure to cold air or rapid changes in ambient temperature, mediated via trigeminal sensory pathways ( Baraniuk & Merck, 2009). This would – at least partially – explain the null result observed trying to induce the PSR in laboratory setting given all the photic sneezes recorded in the real-world.

Despite the inability to artificially elicit sneezes in a controlled setting – which is recognised here as a major limitation of this study – the strong tickling sensations observed suggest that the experimental paradigm is promising with further refinement, as these sensations constitute a physiological response consistent with sneezing, while not being strictly independent from each other. Light stimuli parameters may need more fine-tuning with respect to physiological properties of the human visual system, but the literature reports of sneezing from exposure to artificial light ( Trinkl et al., 2025) reinforce the idea that sneezing can be induced with the present setup.

Our light source uses pulse width modulation (PWM), in which the “phantom array”, a spatial pattern observed by some individuals during saccades, may have been a distraction for the subject and interfered with the evaluation of the bright light by generating coloured fringes due to differences in PWM between the different primaries ( Hershberger & Jordan, 1998; Roberts & Wilkins, 2013). Our participant did not report any distinct visual phenomena consistent with the phantom array effect. The light source also did not provide any stimulation below 400 nm, which introduces a perhaps important difference with the spectral properties of daylight containing energy in the ultra-violet (UV) range that could have played a role in inducing the PSR. Indeed, keratitis and other forms of corneal irritation have been linked to stronger photic sneezing responses ( Askenasy, 1990), presumably via increased trigeminal excitability, and UV light can cause corneal irritation. Future studies should investigate the potential role of UV exposure in inducing the photic sneeze.

## Conclusion

The knowledge presented here contributes to future research by suggesting an approach to bridge the gap between real-world observations – omnipresent in the existing related literature – and the mechanistic understanding of the neural mechanisms of the PSR, which has been related to physiological phenomena such as photophobia, migraine or the trigeminocardiac reflex ( Sasayama et al., 2019; Chowdhury et al., 2019), demonstrating clinical relevance. The establishment of a protocol for consistent PSR induction will open the door to more thorough investigation of these conditions, including defining the biological receptors at play as well as an action spectrum for PSR. Future studies should investigate the variability in individual PSR responses to natural and artificial stimuli, define absolute light intensity thresholds for generating tickling and sneezing responses in photic as well as non-photic sneezers, and more generally attempt to identify the underlying genetic, neurological, and sensory mechanisms of the PSR. Expanding the study to a larger cohort is critical to understand phenotypic variation and interindividual differences in the PSR.

## Declarations

### Ethics approval and consent to participate

This study was reviewed by the Ethics Committee of the Technical University of Munich (2024-74-W-SB).

## Consent for publication

The participant gave informed consent in written form.

## Data Availability

All code and data are available:
• Data: https://doi.org/10.17617/3.LO8EXZ
○ Dataset for Bickerstaff et al., 2025 (DOI: 10.1101/2024.12.11.627890), containing pupillary data, subjective reporting data, light exposure and sneeze logging data and associated metadata. Data is provided in two forms: a full dataset containing all raw and exported data, and a exports-only dataset.
• Code: https://github.com/tscnlab/BickerstaffEtAl_F1000Research_2025

○ Code repository for Bickerstaff et al., 2025 (DOI: 10.1101/2024.12.11.627890), containing a reproducible Python pipeline for generating the outcomes (analysis results and figures) presented in the manuscript.

## Preprint

This manuscript was preprinted on bioRxiv ( Bickerstaff et al., 2024).
