# Supplementary material for: Sneezing in response to naturalistic bright light exposure
Source: F1000Res. 2026 Mar 20;14:1134. Originally published 2025 Oct 17. [Version 2] doi: 10.12688/f1000research.167964.2 (PMC13133618; doi:10.12688/f1000research.167964.2)
Supplement: Peer review history

# Reviewer report: Reviewer response for version 2

**DOI:** 10.5256/f1000research.196705.r473896

**Published:** 2026-04-30

**Authors:** Enrique Germany Morrison

**Affiliations:** 1 Université Catholique de Louvain, Bruxelles, Belgium

General Comment:

 The Case-Report Manuscript is interesting, the design is original, and the naturalistic (Real Case Scenario) in addition to laboratory pairing is a legitimate contribution for a poorly standardized phenomenon. The structure of the manuscript and supporting figures are well constructed and easy to follow. I would just propose some comments linked to minor modifications/additions for providing stronger, reproducible conditions of the reported test settings. Following there are some brief section by section comments.

 Methods Section:

 1) The statistical analysis is not sufficiently specified in the Methods. The Results mention independent t-tests, Bonferroni correction, geometric means, log10 ratios, paired t-test, and exclusion of one outlier, but the Methods do not define the analytic pipeline with enough precision. I would suggest adding a discrete “Statistical analyses” subsection. It should state: whether illuminance was log-transformed before hypothesis testing; how the 100 random non-sneeze windows were sampled; whether windows could overlap each other or occur adjacent to sneeze events; what constituted the outlier and whether its exclusion was prespecified; why a paired t-test was chosen; and how multiple testing was handled across the 20-minute peri-event trajectory. Without this, the analysis is not fully reproducible.

 2) You should define exactly what counted as one “event,” how multiple sneezes within one event were grouped, whether a minimum inter-event interval existed, and whether the timestamp was entered contemporaneously or retrospectively. Because the participant is also an author and knew the study aim, the self-report procedure is particularly important and should be described more rigorously. A brief note on instruction/familiarization is still warranted, even if the participant was the investigator.

 3) Real-world light measurement should be described and stated as a torso-based proxy for corneal illuminance, not a near-equivalent. The current wording says this configuration offered “a good compromise between accurate measurements” and corneal-plane alignment, but this is optimistic. The later discussion rightly acknowledges positional and directional mismatch. That limitation should be foreshadowed in Methods, not introduced only after the fact.

 4) You should specify the exact number of one-shot trials and 30-minute sessions, the total number of stimuli per illuminance level, the order/randomization of intensity presentation, adaptation conditions before the 30-minute protocol, fixation instructions, eye-tracking preprocessing, blink handling, pupil metric used for analysis, and room/environmental conditions including approximate ambient temperature. Given that the discussion invokes UV absence, PWM, and thermal stability as plausible reasons for null induction, the laboratory stimulus description should be correspondingly more detailed.

 Results Section:

 5) The laboratory null result needs stronger numerical reporting. State plainly: “0 of >150 stimuli induced sneezing.” Then provide at least one compact sentence with tickle ratings by illuminance level and paradigm, for example median or mean ± SD per level. Right now, the claim of a monotonic relationship is visually plausible from Figure 2, but it is not numerically anchored in the text and no formal statistical test is reported for pupil or tickle outcomes.

 6) Also, the abstract’s “peaking 2 minutes prior” should either be explicitly quantified in the main text or softened in the abstract.

 Conclusion Section:

 7) The treatment of tickle is also slightly overstated. It is reasonable to interpret high tickle ratings as evidence of partial engagement of whatever pathway leads toward sneezing, but that remains inferential. It is not demonstrated that nasal tickle in this setup is a validated surrogate endpoint for PSR. The paper should say so directly. A precise formulation would be: “Tickle ratings may represent a proximal subjective correlate of the response, but they cannot be assumed to be equivalent to sneeze induction.”

Is the case presented with sufficient detail to be useful for teaching or other practitioners?

Partly

Is the work clearly and accurately presented and does it cite the current literature?

Yes

If applicable, is the statistical analysis and its interpretation appropriate?

Partly

Are all the source data underlying the results available to ensure full reproducibility?

Yes

Are the conclusions drawn adequately supported by the results?

Partly

Is the background of the case’s history and progression described in sufficient detail?

Yes

Reviewer Expertise:

Biomedical Engineering, Neuroscience, Data and Signal Processing, Sensors.

I confirm that I have read this submission and believe that I have an appropriate level of expertise to confirm that it is of an acceptable scientific standard, however I have significant reservations, as outlined above.

# Reviewer report: Reviewer response for version 2

**DOI:** 10.5256/f1000research.196705.r469324

**Published:** 2026-03-24

**Authors:** Zacharias Kalentakis

**Affiliations:** 1 National and Kapodistrian University of Athens, Athens, Greece

Congratulations on your work, *“Sneezing in Response to Naturalistic Bright Light Exposure.”* The study is well-structured and thoughtfully designed, offering valuable insights into a relatively underexplored topic. All suggested revisions have been thoroughly addressed.

Is the case presented with sufficient detail to be useful for teaching or other practitioners?

Partly

Is the work clearly and accurately presented and does it cite the current literature?

Yes

If applicable, is the statistical analysis and its interpretation appropriate?

Partly

Are all the source data underlying the results available to ensure full reproducibility?

Partly

Are the conclusions drawn adequately supported by the results?

No

Is the background of the case’s history and progression described in sufficient detail?

Partly

Reviewer Expertise:

Otorhinolaryngology, Rhinology

I confirm that I have read this submission and believe that I have an appropriate level of expertise to confirm that it is of an acceptable scientific standard.

# Reviewer report: Reviewer response for version 1

**DOI:** 10.5256/f1000research.185114.r437041

**Published:** 2026-01-05

**Authors:** Zacharias Kalentakis

**Affiliations:** 1 National and Kapodistrian University of Athens, Athens, Greece

The article presents a very clever approach to studying PSR under controlled laboratory conditions. However, several aspects of the manuscript need clarification or expansion before it can be considered for publication:

1. **Background / Introduction**
  - The background section should provide more detailed information on the photic sneeze reflex (PSR), including its underlying pathophysiology. In particular, it should be clearly explained how sudden exposure to light can trigger the reflex.
  - A more thorough review of the relevant literature on PSR would strengthen the rationale for the study.
2. **Methods**
  - The methodology should clarify why the data were collected in 2022 while the manuscript was only submitted in 2025. Any delays or reasons (e.g. additional analyses, interruptions, etc.) should be briefly explained.
  - It should be stated whether and how the study participant was trained or instructed regarding PSR, especially given that they were asked to complete questionnaires that are central to the research data. Details on instructions, familiarization procedures, or pilot testing would be useful.
  - The manuscript does not mention whether the participant has any relevant medical history. It should be specified whether the participant underwent clinical evaluation by an ENT specialist, ophthalmologist, and/or neurologist prior to inclusion, and whether any conditions that could influence PSR were excluded.
3. **Results**
  - The results section should provide a more detailed quantitative presentation of the data, rather than relying mainly on diagrams. Numerical values, measures of variability, and any statistical tests performed should be clearly described in the text.
  - The observation that exposure to bright light induces the reflex is somewhat expected; however, the isolated observation that the reflex was triggered once when light levels were *reduced* requires stronger support and discussion. This finding should be substantiated with more detailed data and, if possible, a plausible explanation or hypothesis.
4. **Main Limitation: Failure to Elicit PSR**
  - The experiments did not succeed in reliably eliciting PSR. This is a major limitation of the study and should be explicitly acknowledged and discussed. The authors should comment on possible reasons (e.g. stimulus parameters, individual variability, experimental setup) and how future studies might address this issue.
5. **Discussion and Limitations**
  - The manuscript essentially lacks dedicated Discussion and Limitations sections. These should be added.
  - The Discussion should interpret the findings in the context of existing literature, explain the implications of both the positive and negative results, and address the unexpected observations (e.g. reflex on light reduction).
  - A Limitations paragraph should summarize the main methodological and conceptual constraints of the study (single participant, failure to elicit reflex, lack of comprehensive clinical evaluation if applicable, etc.).
6. **Conclusion and Future Directions**
  - The conclusion should elaborate on how the attempted experimental induction of PSR can contribute to future research, even if the primary aim was not fully achieved.
  - It would be particularly interesting to investigate and report the minimum light intensity required to trigger PSR and to compare this threshold across multiple individuals. Even if this was not done in the present study, it could be proposed as a concrete direction for future work.
  - The authors should also clarify that the sensation of a tickle in the nose does not necessarily lead to a sneeze, and that not every sneeze is preceded by such a sensation. This distinction is important for interpreting subjective reports and questionnaire data.
7. **References**
  - The reference list would benefit from the inclusion of more recent and comprehensive bibliographic sources on PSR and related reflexes, to ensure that the manuscript is aligned with current knowledge.

Overall, the study concept is original and promising, but the manuscript needs substantial revision in terms of methodological clarity, data presentation, discussion of findings, and integration with up-to-date literature.

Is the case presented with sufficient detail to be useful for teaching or other practitioners?

Partly

Is the work clearly and accurately presented and does it cite the current literature?

Yes

If applicable, is the statistical analysis and its interpretation appropriate?

Partly

Are all the source data underlying the results available to ensure full reproducibility?

Partly

Are the conclusions drawn adequately supported by the results?

No

Is the background of the case’s history and progression described in sufficient detail?

Partly

Reviewer Expertise:

Otorhinolaryngology, Rhinology

I confirm that I have read this submission and believe that I have an appropriate level of expertise to confirm that it is of an acceptable scientific standard, however I have significant reservations, as outlined above.

# Response

**Published:** 2026-02-16

**Authors:** Manuel Spitschan

**Affiliations:** Not Applicable, Germany

We thank the reviewer for their insightful comments, which we respond to below.

***Background / Introduction***

***The background section should provide more detailed information on the photic sneeze reflex (PSR), including its underlying pathophysiology. In particular, it should be clearly explained how sudden exposure to light can trigger the reflex.***

***A more thorough review of the relevant literature on PSR would strengthen the rationale for the study.***

 We have expanded our discussion of theories regarding the pathophysiology of the PSR, citing relevant literature, and added text to further justify the rationale for the study (i.e. absence of evidence despite attempts through experimental approaches)

***Methods***

***The methodology should clarify why the data were collected in 2022 while the manuscript was only submitted in 2025. Any delays or reasons (e.g. additional analyses, interruptions, etc.) should be briefly explained.***

 We have added a sentence describing gap between data collection and publication

***It should be stated whether and how the study participant was trained or instructed regarding PSR, especially given that they were asked to complete questionnaires that are central to the research data. Details on instructions, familiarization procedures, or pilot testing would be useful.***

 It is already mentioned that author LB of the manuscript is the subject of the case report, implying that the participant was aware and trained regarding the experimental methods.

***The manuscript does not mention whether the participant has any relevant medical history. It should be specified whether the participant underwent clinical evaluation by an ENT specialist, ophthalmologist, and/or neurologist prior to inclusion, and whether any conditions that could influence PSR were excluded.***

 We have added text to describe the medical history of the subject and absence of clinical assessment

***Results***

***The results section should provide a more detailed quantitative presentation of the data, rather than relying mainly on diagrams. Numerical values, measures of variability, and any statistical tests performed should be clearly described in the text.***

 We expanded and restructured the results section significantly, adding more quantitative descriptions of the real-world light exposure data. Added geometric means and standard deviations for the light exposure data (Figure 1B). Added statistical tests performed.

**The observation that exposure to bright light induces the reflex is somewhat expected; however, the isolated observation that the reflex was triggered once when light levels were *reduced* requires stronger support and discussion. This finding should be substantiated with more detailed data and, if possible, a plausible explanation or hypothesis.**

 We now describe and discuss the 7/82 cases where light decreased and a photic sneeze was recorded. After having another look at the data, it was found that more than one observation had this characteristic, which was an oversight.

***Main Limitation: Failure to Elicit PSR***

***The experiments did not succeed in reliably eliciting PSR. This is a major limitation of the study and should be explicitly acknowledged and discussed. The authors should comment on possible reasons (e.g. stimulus parameters, individual variability, experimental setup) and how future studies might address this issue.***

 “The authors should comment on possible reasons (e.g. stimulus parameters, individual variability, experimental setup)…”: This is addressed in the discussion and limitations section; “…and how future studies might address this issue.”: Lines 208 to 220: This is addressed in the conclusion section

***Discussion and Limitations***

***The manuscript essentially lacks dedicated Discussion and Limitations sections. These should be added.***

***The Discussion should interpret the findings in the context of existing literature, explain the implications of both the positive and negative results, and address the unexpected observations (e.g. reflex on light reduction).***

***A Limitations paragraph should summarize the main methodological and conceptual constraints of the study (single participant, failure to elicit reflex, lack of comprehensive clinical evaluation if applicable, etc.).***

 These points are now addressed by adding a joint Discussion and Limitations section

***Conclusion and Future Directions***

***The conclusion should elaborate on how the attempted experimental induction of PSR can contribute to future research, even if the primary aim was not fully achieved.***

 We now describe the clinical relevancy of studying the PSR

***It would be particularly interesting to investigate and report the minimum light intensity required to trigger PSR and to compare this threshold across multiple individuals. Even if this was not done in the present study, it could be proposed as a concrete direction for future work.***

 We have added text to suggest investigating thresholds for PSR induction

***The authors should also clarify that the sensation of a tickle in the nose does not necessarily lead to a sneeze, and that not every sneeze is preceded by such a sensation. This distinction is important for interpreting subjective reports and questionnaire data.***

 We have made it clear that tickles and sneezes are linked while not independent from each other

***References***

***The reference list would benefit from the inclusion of more recent and comprehensive bibliographic sources on PSR and related reflexes, to ensure that the manuscript is aligned with current knowledge.***

 The Trinkl et al., 2025 mini-review is the most up-to-date reference on the topic of PSR and is referenced multiple times in the case report. This case report only references critical and relevant literature.

# Reviewer report: Reviewer response for version 1

**DOI:** 10.5256/f1000research.185114.r427886

**Published:** 2025-11-12

**Authors:** Arnold Wilkins

**Affiliations:** 1 Department of Psychology, University of Essex, Colchester, UK

Given that the experimenters were unsuccessful in inducing sneezing in the lab by light stimulation one is led to wonder what the important differences between lab conditions and the conditions under which sneezing typically occurs.  These may be characterised as reflecting differences in the individual's physiology and differences in the light stimulation. As regards the individual's physiology, thermal comfort is one obvious difference between the natural and lab conditions. Sneezing is usually occasioned when an individual feels the cold, something they are more likely to do when outside than indoors. We are told that "Sneezing often took place during transitions between environments, e.g., walking from home to the bus station, or from the bus station to the workplace." Presumably the indoor temperature was stable and comfortable, unlike the conditions prevalent when sneezing occurred. Thermal instability is therefore a consideration.  There were also important differences between the light used in the lab and the daylight outside.  The multiLED source did not provide stimulation below 400nm.  It is possible that the UV present outdoors contributed to sneezing, given that Askenasy notes that keratitis has been reported to enhance the photic sneeze reflex.  If LB wears glasses this is unlikely to be an important concern given that CR39 lenses filter out most light below 380nm. If LB does not, however, there is the (admittedly remote) possibility that UV played a role.  Another indirect concern is that the light engine used pulse width modulation (PWM). Some individuals can discriminate steady from intermittent light at frequencies exceeding 15kHz.  Those that can do so tend to suffer eye-strain in everyday life. The intermittent light generates a spatial pattern during a saccade known (unfortunately) as the phantom array. It is remotely possible that the phantom array interfered in some way with eye movements or with appreciation of the bright light. If coloured fringes were visible during a saccade owing to the differences in PWM between the various LED sources, this would have been a distraction, if nothing more.

Is the case presented with sufficient detail to be useful for teaching or other practitioners?

Partly

Is the work clearly and accurately presented and does it cite the current literature?

Yes

If applicable, is the statistical analysis and its interpretation appropriate?

Not applicable

Are all the source data underlying the results available to ensure full reproducibility?

Partly

Are the conclusions drawn adequately supported by the results?

No

Is the background of the case’s history and progression described in sufficient detail?

Partly

Reviewer Expertise:

vision science, migraine, epilepsy

I confirm that I have read this submission and believe that I have an appropriate level of expertise to confirm that it is of an acceptable scientific standard, however I have significant reservations, as outlined above.

# Response

**Published:** 2026-02-16

**Authors:** Manuel Spitschan

**Affiliations:** Not Applicable, Germany

We thank the reviewer for their insightful comments, which we address below.

***Given that the experimenters were unsuccessful in inducing sneezing in the lab by light stimulation one is led to wonder what the important differences between lab conditions and the conditions under which sneezing typically occurs.  These may be characterised as reflecting differences in the individual's physiology and differences in the light stimulation. As regards the individual's physiology, thermal comfort is one obvious difference between the natural and lab conditions. Sneezing is usually occasioned when an individual feels the cold, something they are more likely to do when outside than indoors. We are told that "Sneezing often took place during transitions between environments, e.g., walking from home to the bus station, or from the bus station to the workplace." Presumably the indoor temperature was stable and comfortable, unlike the conditions prevalent when sneezing occurred. Thermal instability is therefore a consideration.***

 We have now detailed what effects temperature could have and cited relevant literature.

***There were also important differences between the light used in the lab and the daylight outside.  The multiLED source did not provide stimulation below 400nm.  It is possible that the UV present outdoors contributed to sneezing, given that Askenasy notes that keratitis has been reported to enhance the photic sneeze reflex.  If LB wears glasses this is unlikely to be an important concern given that CR39 lenses filter out most light below 380nm. If LB does not, however, there is the (admittedly remote) possibility that UV played a role.***

 We have now added medical history and clinical evaluation of participant, and also detailed what effects UV could have and cited relevant literature.

***Another indirect concern is that the light engine used pulse width modulation (PWM). Some individuals can discriminate steady from intermittent light at frequencies exceeding 15kHz.  Those that can do so tend to suffer eye-strain in everyday life. The intermittent light generates a spatial pattern during a saccade known (unfortunately) as the phantom array. It is remotely possible that the phantom array interfered in some way with eye movements or with appreciation of the bright light. If coloured fringes were visible during a saccade owing to the differences in PWM between the various LED sources, this would have been a distraction, if nothing more.***

 We have now detailed what the phantom array is, what effects it could have and cited relevant literature.
